# Metabolic models predict bacterial passengers in colorectal cancer

**DOI:** 10.1186/s40170-020-0208-9

**Published:** 2020-02-10

**Authors:** Daniel R. Garza, Rahwa Taddese, Jakob Wirbel, Georg Zeller, Annemarie Boleij, Martijn A. Huynen, Bas E. Dutilh

**Affiliations:** 1 Centre for Molecular and Biomolecular Informatics, Radboud University Medical Centre, Postbus 9101, 6500 HB Nijmegen, The Netherlands; 2 Department of Pathology, Radboud University Medical Center, Postbus 9101, 6500 Nijmegen, HB Netherlands; 3 European Molecular Biology Laboratory, Structural and Computational Biology Unit, 69117 Heidelberg, Germany; 4 Theoretical Biology and Bioinformatics, Sience4Life, Utrecht University, Hugo R. Kruytgebouw, Room Z-509, Padualaan 8, Utrecht, The Netherlands

**Keywords:** Genome-scale metabolic models, Colorectal cancer microbiome, Colorectal cancer metabolome, Bacterial driver-passenger model

## Abstract

**Background:**

Colorectal cancer (CRC) is a complex multifactorial disease. Increasing evidence suggests that the microbiome is involved in different stages of CRC initiation and progression. Beyond specific pro-oncogenic mechanisms found in pathogens, metagenomic studies indicate the existence of a microbiome signature, where particular bacterial taxa are enriched in the metagenomes of CRC patients. Here, we investigate to what extent the abundance of bacterial taxa in CRC metagenomes can be explained by the growth advantage resulting from the presence of specific CRC metabolites in the tumor microenvironment.

**Methods:**

We composed lists of metabolites and bacteria that are enriched on CRC samples by reviewing metabolomics experimental literature and integrating data from metagenomic case-control studies. We computationally evaluated the growth effect of CRC enriched metabolites on over 1500 genome-based metabolic models of human microbiome bacteria. We integrated the metabolomics data and the mechanistic models by using scores that quantify the response of bacterial biomass production to CRC-enriched metabolites and used these scores to rank bacteria as potential CRC passengers.

**Results:**

We found that metabolic networks of bacteria that are significantly enriched in CRC metagenomic samples either depend on metabolites that are more abundant in CRC samples or specifically benefit from these metabolites for biomass production. This suggests that metabolic alterations in the cancer environment are a major component shaping the CRC microbiome.

**Conclusion:**

Here, we show with in sillico models that supplementing the intestinal environment with CRC metabolites specifically predicts the outgrowth of CRC-associated bacteria. We thus mechanistically explain why a range of CRC passenger bacteria are associated with CRC, enhancing our understanding of this disease. Our methods are applicable to other microbial communities, since it allows the systematic investigation of how shifts in the microbiome can be explained from changes in the metabolome.

## Background

Colorectal cancer (CRC) is the third leading cancer worldwide and more than 1.2 million new cases are diagnosed each year, approximately 45% of which are fatal [1, 2]. CRC is a complex multifactorial disease with many risk factors statistically and mechanistically associated with its incidence and prevalence, including host genetics, smoking, excessive alcohol consumption, high consumption of red and processed meat, obesity, and diabetes [3–7]. Many recent studies have highlighted possible roles of the gut microbiome in the initiation and progression of CRC (for reviews, see [8–13]). Additionally, many of the factors that are associated with CRC development are also associated with possible shifts in the composition of the microbiome, such as the aforementioned dietary factors [14].

Dietary compounds, the resident microbiota, and their secreted products are among the most significant external components that interact with gut epithelial cells at the mucosal surface [8]. Under certain conditions, gut bacteria can favor tumorigenesis by promoting inflammation, DNA damage, cell proliferation, or anti-apoptotic signaling [9–11]. Several specific bacterial mechanisms that can trigger cancer initiation or progression have been identified by cell and animal studies. For instance, the commensal *Enterococcus faecalis* bacteria produces extracellular superoxide, which can induce DNA damage, chromosomal instability, and malignant transformation in mammalian cells [15]. There are many other specific cancer-driving mechanisms associated with bacteria that are commonly found in the human gut, such as *Helicobacter pylori* [16], enterotoxigenic *Bacteroides fragilis* [17], and colibactin-producing *Escherichia coli* [18].

Besides specific causal mechanisms, collective effects of the microbiome community have been associated with CRC, generally termed dysbiosis. For instance, in a mouse model of CRC, specific-pathogen-free (SPF) C57BL/6 mice developed significantly fewer tumors under germ-free conditions [19], which was also observed when these mice were treated with broad-spectrum antibiotics [20]. Conversely, these mice developed significantly more tumors when fed with stool from CRC patients, compared to mice fed with stool from healthy controls [21].

Certain microbiome community profiles have been associated with CRC in humans. Metagenomic studies have found consistent similarities in microbial communities derived from the tumor site of different patients compared to the healthy tissue [22, 23] and specific bacterial taxa have been consistently associated with stool samples of CRC patients [24–28]. This CRC microbiome signature is suggested to be an important feature for the early diagnosis of CRC [24].

The evidence described above that links the microbiome to CRC suggests a complex interaction that is influenced by many different factors. In contrast to other microbe-induced cancers [29], CRC has not been associated with a single microbial species or mechanism and is understood to result from cumulative host and microbial factors [9]. A conceptual model to explain the shifts in the CRC microbiome is the “bacterial driver-passenger model” [11], which describes a chronological order in the association of different bacteria with CRC. According to this model, “driver bacteria” first cause DNA damage and promote the malignant transformation of epithelial stem cells and, after tumorigenesis is initiated, this process promotes niche alterations that favor the outgrowth of “passenger bacteria”. These bacteria may or may not further aggravate the progression of the disease and are generally found to be enriched in the microbiome of CRC patients [11].

In this study, we implemented a computational approach to answer the question whether the outgrowth of CRC associated bacteria can be explained by changes in CRC metabolites, as expected from the driver-passenger model. For this purpose, we analyzed the data from five metagenomic case-control studies [24–28] and 35 metabolomic studies [30–64] to identify specific bacteria and metabolites that are enriched in CRC patients. We used over 1500 genome-scale metabolic models (GSMMs) from human-associated bacterial strains [65] and found that CRC enrichment can be predicted from bacterial dependency on CRC metabolites and from the specific growth advantage conferred by these metabolites. We thus linked metagenomic and metabolomic data with mechanistic models that explain why a range of bacteria are specifically enriched in the CRC tumor environment.

## Results

**Fig. 1:**
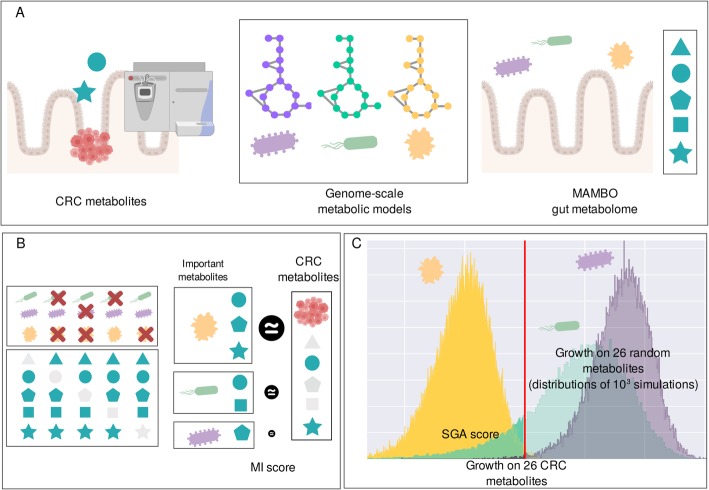
Computational approach to identify colorectal cancer metabolic passengers. **a** As inputs we used (i) CRC metabolites that were identified from metabolomics literature, (ii) genome-scale metabolic models, and (iii) a basal gut-like environment [66]. **b** Important metabolites for biomass production were defined as the ones that reduced growth if that metabolite was removed. The MI score was defined by comparing the list of important metabolites with the CRC metabolites. **c** Specific growth advantage was evaluated by supplementing the basal environment with the 26 CRC metabolites, and comparing this with the growth advantage on 1000 sets of 26 random metabolites. The SGA score was defined as the proportion of random sets where the growth advantage was lower than with the CRC metabolites (depicted in the distribution mass to the left of the red vertical line that indicates growth on the CRC metabolites). In the illustrated examples, the yellow bacteria is predicted to be a CRC passenger

We set out to identify bacteria that respond to the altered metabolic profile in the CRC tumor microenvironment [11]. Our approach is illustrated in Fig. 1. In summary, we first identified CRC metabolites that are enriched in the tumor environment versus healthy tissue as measured by at least three metabolomic studies [30–64] (Fig. 1a, Table 1). To evaluate the effect of CRC metabolites on human microbiome bacteria, we used 1544 genome-scale metabolic models (GSMMs) derived from the human microbiome that allow bacterial growth to be mechanistically modeled in silico in a well-defined metabolic environment resembling the human intestinal lumen [65] (Fig. 1a). This environment is referred to in the text as the “MAMBO” environment. We also reproduced all of the in silico experiments using two alternative metabolic compositions as basal environments which are referred to as “Western diet” and “high-fiber diet” environments [66]. For the specific composition of the basal environments, see Additional file 1: Table S1. We then used computational experiments to integrate information about metabolite enrichment in CRC with mechanistic models and to rank bacteria as potential CRC passengers (Fig. 1b, c). These experiments are further explained in the next subtopics.

**Table 1:** Metabolites enriched or depleted in CRC

Metabolite	References
L-Valine	Enriched [33, 35, 41, 62, 64]
Stearic acid	Enriched [31, 34, 44, 62]
L-Arginine	Enriched [33, 35, 41]
Phenylalanine	Enriched [33, 34, 41, 44]
Spermidine	Enriched [35, 48, 55]
Taurine	Enriched [32, 34, 35, 40–43, 48, 63]
L-Threonine	Enriched [33, 35, 41, 42, 62, 64]
Glutathione	Enriched [35, 41, 47, 64]
Putrescine	Enriched [35, 41, 48]
Palmitic acid	Enriched [31, 34, 44]
Proline	Enriched [33–35, 41, 44, 62]
Asparagine	Enriched [33, 35, 41, 62]
Hypoxanthine	Enriched [33, 35, 44]
Lactic acid	Enriched [31, 32, 34, 35, 40–42, 62, 63]
Aspartic acid	Enriched [30, 33, 35, 41, 42, 47]
Cholesterol	Enriched [32–34, 44, 62]
Glutamic acid	Enriched [33, 35, 41, 42, 44, 47]
Tyrosine	Enriched [33, 35, 44, 63] Depleted [64]
Choline	Enriched [32, 34, 40, 44, 50–52, 63]
Uridine	Enriched [34, 41, 44, 62]
Serine	Enriched [33, 35, 41, 62, 64]
Vaccenic acid	Enriched [31, 34, 44] Depleted [33]
Lysine	Enriched [33, 35, 41, 44]
Glycine	Enriched [31, 33–35, 41, 45, 62] Depleted [32]
Methionine	Enriched [33, 35, 44, 62]
Isoleucine	Enriched [33, 35, 41, 62, 64]
Glucose	Depleted [31, 32, 40–42, 44, 62, 64]
Glutamine	Enriched [64] Depleted [33, 35, 38]
Myoinositol	Depleted [42, 44, 47, 50, 64]

### Individual CRC metabolites show a high overlap with metabolites that promote growth of CRC bacteria

To investigate in which bacteria the CRC metabolites are important for biomass production, we developed a measure that is referred to in the text as the “metabolite importance”, or MI score. The MI score is defined by removing CRC metabolites one by one from the environment of the GSMMs and measuring the impact of the removal on predicted in silico growth (Fig. 1b). The measure is based on the Ochiai similarity score [67], a score commonly used in ecological studies, that presents a range between 0 and 1 (see “Methods” section for details), where 1 means that there is a perfect overlap between the CRC metabolites and the metabolites that are important for growth, while 0 means there are is no overlap.

**Fig. 2:**
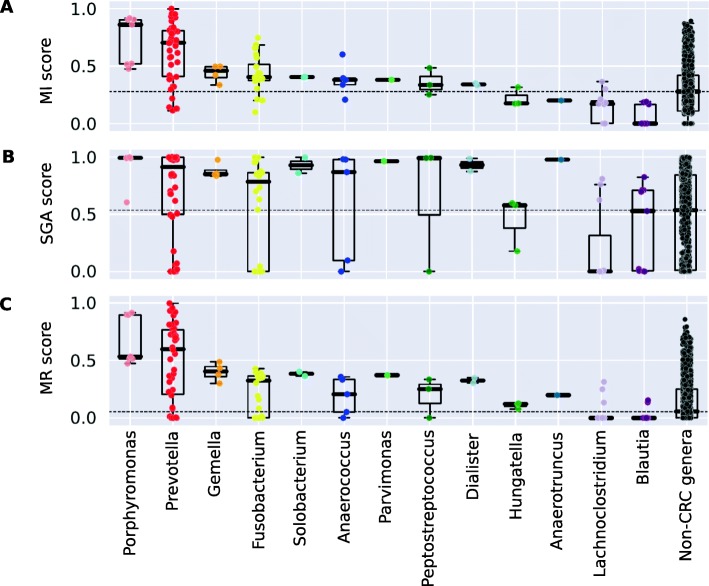
Distribution of the metabolite importance (MI) (**a**), specific growth advantage (SGA) (**b**), and metabolite response (MR) scores (**c**) in CRC and non-CRC bacteria. Each dot represents a GSMM, CRC genera are shown separately while non-CRC genera are combined

**Fig. 3:**
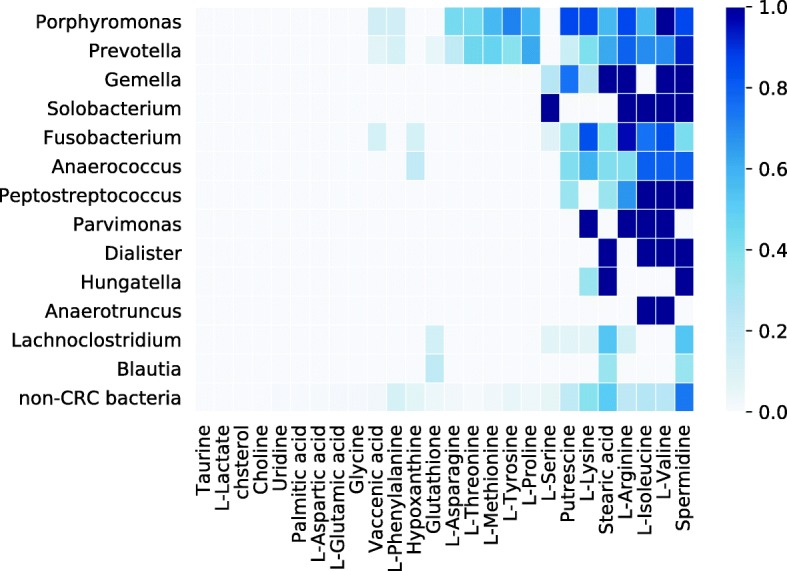
Distribution of important metabolites within CRC and non-CRC bacteria. Each cell is colored according to the fraction of models that require the metabolite for biomass production

We calculated MI scores for all human microbiome bacteria (Additional file 2: Table S2) using the metabolites that are enriched in CRC as identified by our literature search (Table 1). Next, we identified CRC bacteria that are significantly enriched in the metagenomes of CRC patients compared to healthy controls from five metagenomic case-control studies [24–28](Fig. 1b, Table 2). We then evaluated whether the genera containing CRC bacteria have higher MI scores than non-CRC bacteria, which would suggest that CRC metabolites are more important for biomass production in CRC bacteria than in other bacteria. As shown in Fig. 2a, most CRC genera have on average higher MI scores than non-CRC genera (adj. *P*=6.9e-08; Mann-Whitney U test). Fig. 3 summarizes the association of CRC bacterial genera to specific CRC metabolites, showing that different bacteria depend on different groups of CRC metabolites and, in general, CRC bacteria depend on more CRC metabolites than non-CRC bacteria (Fig. 3).

**Table 2:** Bacterial genera enriched in CRC

Genus	Enriched mOTUs	AUC/adj. *p values*
Parvimonas	Parvimonas_micra [1145] Parvimonas_sp._oral_taxon_110 [4961]	0.71/1.8E−20 0.57/3.7E−08
Dialister	Dialister mOTU [0561]	065/5.0E−20
Gemella	Gemella_morbillorum [4513]	0.70/3.E−18
Fusobacterium	Fusobacterium_nucleatum_subsp._animalis_[C] [0776] Fusobacterium_nucleatum_subsp._nucleatum_[C] [0777] Fusobacterium_nucleatum_subsp._vincentii_[C] [0754] Fusobacterium_sp._oral_taxon_370 [1403]	0.66/9.6E−17 0.57/4.4E−08 0.57/2.1E−07 0.56/3.7E−07
Peptostreptococcus	Peptostreptococcus_stomatis [4614]	0.67/8.4E−16
Porphyromonas	Porphyromonas mOTU [2350] Porphyromonas_somerae [2101] Porphyromonas_asaccharolytica [1517] Porphyromonas mOTU [0125] Porphyromonas mOTU [1184] Porphyromonas_uenonis [2102]	0.61/1.6E−13 0.57/5.5E−09 0.58/2.9E−08 0.57/1.1E−07 0.56/8.6E−07 0.59/1.7E−10
Solobacterium	Solobacterium_moorei [0531]	0.64/1.7E−10
Lachnoclostridium	[Clostridium]_symbiosum_[C] [1475]	0.67/2.3E−10
Hungatella	Hungatella_hathewayi [0882]	0.66/3.7E−09
Prevotella	Prevotella_intermedia [0515] Prevotella_nigrescens [0276]	0.58/2.8E−09 0.56/5.5E−08
Anaerococcus	Anaerococcus_sp._[C_obesiensis/vaginalis] [0429]	0.58/6.6E−07
Blautia	[Ruminococcus]_torques_[C] [1376]	0.64/1.5E−07
Anaerotruncus	Anaerotruncus mOTU [1529]	0.60/1.0E−06

### The combination of CRC metabolites confers specific growth advantage for CRC bacteria

We next tested which bacteria showed a specific response to the increased availability of combined CRC metabolites in the context of the gut environment. For this purpose, we developed the “specific growth advantage,” or SGA score that evaluates how an increased growth rate of a GSMM depends on supplementing the environment with a specific set of metabolites. In general, many bacterial models respond to increased availability of metabolites with increased growth (not shown), so to quantify whether a strain responded specifically to enrichment of CRC metabolites, we compared this growth advantage to the growth advantage when random metabolite subsets were enriched (Fig. 1c). The SGA score between 0 and 1 consists of the proportion of random sets of enriched metabolites that caused a smaller growth advantage than when the CRC metabolites were enriched. Based on the supplementation of all CRC metabolites at once, this score is complementary to the MI score, which is based on depletion of individual metabolites. The results were consistent with the MI score, as the average SGA score was significantly higher for CRC bacteria than for non-CRC bacteria (adj. *p* = 4.6E−5; Mann-Whitney *U* test) (Fig. 2b).

Significantly higher MI and SGA scores for CRC bacteria than for non-CRC bacteria (above) indicate that these bacteria benefit from the CRC metabolites in the tumor microenvironment. Both scores reflect different but related aspects of the association between the CRC metabolites and bacterial metabolism and are thus weakly but significantly correlated (Spearman correlation 0.12, *p* = 2.4 E−7). We combined the two scores into a single score by using a copula function that accounts for this correlation. We refer to the combined score in the rest of the text as the “metabolite response” or MR score. As shown in Fig. 2c, the MR-score was significantly higher for CRC bacteria than for non-CRC bacteria (*p =* 3.9E−7; Mann-Whitney *U* test).

### Bacteria that profit from CRC metabolites are enriched in CRC

**Fig. 4:**
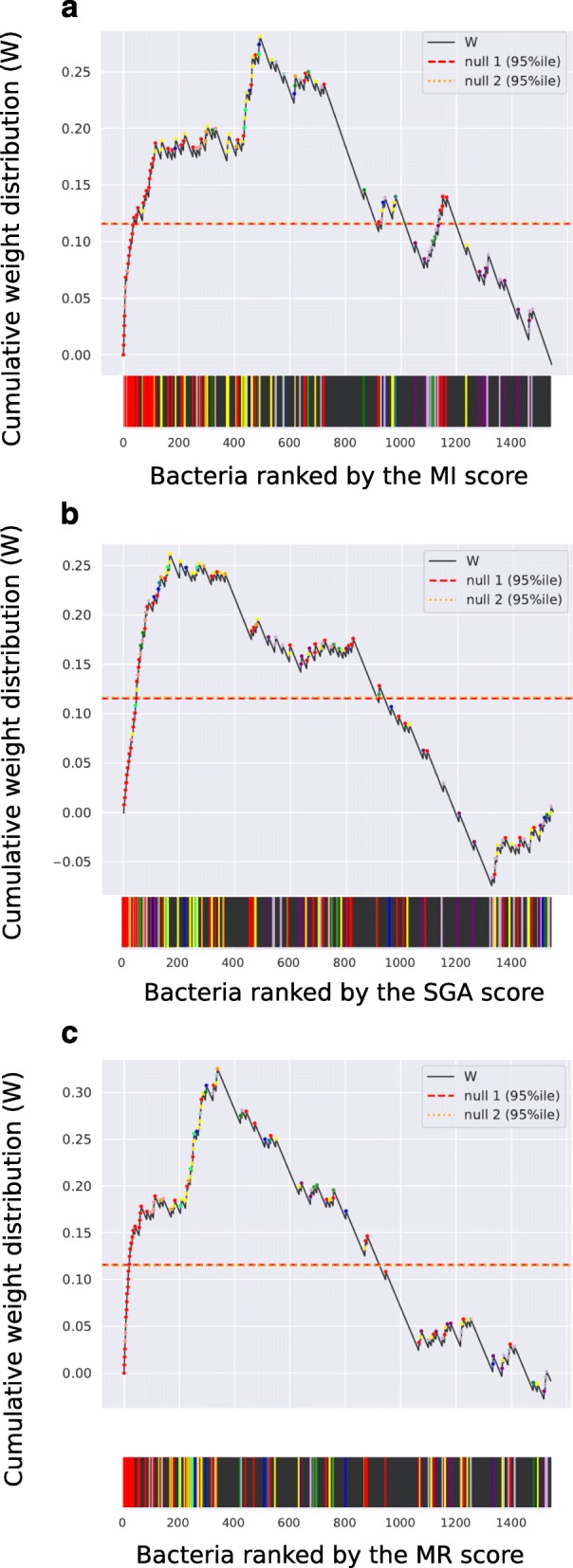
Cumulative weight distribution *W* of bacteria ranked by the MI (**a**), SGA (**b**), and MR (**c**) scores. Each increase in W is linked to a colored dot and corresponding vertical line in the color strips, representing GSMMs belonging to a CRC genera. Non-CRC bacteria are represented by a black vertical line and an associated decrease in *W*. Null 1 indicates the 95 percentile of the maximum cumulative weight distribution in 10^4^ randomizations of the model rankings in the list. Null 2 is the 95 percentile of the maximum cumulative weight distribution in 10^4^ weighted randomizations of the CRC-association of genera

Above, we showed that bacterial genera that are enriched in CRC tend to have higher average MI, SGA, and MR scores than other genera. We next evaluated whether CRC bacteria are ranked significantly higher than other bacteria in a ranked list based on our scores. This would indicate that our ranking is enriched for CRC bacteria as a group compared to non-CRC bacteria and suggest that metabolic alterations in the CRC environment can systematically explain the differential abundance measured by metagenomes. For this purpose, we generated a cumulative weight distribution curve (W) by iterating over the lists ranked by our scores from top to bottom. W was increased by a normalized constant (see “Methods” section) if the bacterium was found to be enriched in CRC and decreased otherwise. As shown in the color strips of Fig. 4, CRC bacteria ranked high on the lists for all three scores and the cumulative weight curve *W* is mostly increasing with the first bacteria. This implies that the top bacteria are mostly from genera that are found by metagenomics to be enriched in CRC. Importantly, these enrichments are significantly higher than expected based on two related null hypotheses: (1) random shuffling of the bacterial labels in the list ranked by our scores and (*p* < 1.0E−4) (2) random shuffling of the labels for CRC-enriched bacterial genera (*p* < 1.0E−4), as shown by the curves *W* surpassing the horizontal 95 percentiles of the peak values of 10^4^ simulations with the null distributions (Fig. 4a–c, Table 3). Enrichment for CRC bacteria improves when using the MR score, which combines the MI and SGA scores, compared to using any of the scores individually. This is shown by a greater maximum value of the cumulative weight curve for the MR score (Fig. 4) and indicates that both MI and SGA scores provide complementary information about the enrichment of CRC bacteria in the tumor microenvironment.

**Table 3:** Enrichment for CRC bacteria in different basal environments and model subsets

Basal environment	Model subset	Score	Max *W*	CRC enrich. *p* value (null 1)	CRC enrich. *p* value (null 2)	Mann-Whitney *U* statistic	Mann-Whitney *p* value	Adj. *p* value^#^
MAMBO	All	*MI*	0.2813	1.00E−04	1.00E−04	109720.5	1.79E−08	*6.90E−08*
		*SGA*	0.2620	1.00E−04	1.00E−04	103113	2.20E−05	*4.57E−05*
		*MR*	0.3255	1.00E−04	1.00E−04	107855.5	1.14E−07	*3.85E−07*
	Gut	*MI*	0.3995	1.00E−04	1.00E−04	10156	2.09E−05	*4.57E−05*
		*SGA*	0.3330	1.00E−04	1.00E−04	8694	1.98E−02	*2.97E−02*
		*MR*	0.4258	2.00E−04	1.00E−04	9491	5.28E−04	*1.02E−03*
	AGORA	*MI*	0.4302	1.00E−04	1.00E−04	43996	1.53E−15	*1.38E−14*
		*SGA*	0.3446	1.00E−04	1.00E−04	38949	1.61E−07	*4.83E−07*
		*MR*	0.4423	1.00E−04	1.00E−04	42642	2.51E−13	*1.69E−12*
Western diet	All	*MI*	0.3193	1.00E−04	1.00E−04	113411.5	1.44E−10	*6.48E−10*
		*SGA*	0.1035	9.16E−02	9.74E−02	86876.5	2.73E−01	2.73E−01
		*MR*	0.1327	2.17E−02	1.94E−02	91885	3.82E−02	5.16E−02
	Gut	*MI*	0.4713	1.00E−04	1.00E−04	10493	2.57E−06	*6.31E−06*
		*SGA*	0.2607	1.36E−02	1.59E−02	8417.5	4.27E−02	5.49E−02
		*MR*	0.3544	5.00E−04	3.00E−04	9265.5	9.35E−04	*1.68E−03*
	AGORA	*MI*	0.4231	1.00E−04	1.00E−04	44395.5	2.82E−16	*3.81E−15*
		*SGA*	0.1744	8.80E−03	8.70E−03	31390.5	1.16E−01	1.36E−01
		*MR*	0.2412	1.00E−04	1.00E−04	34690.5	1.01E−03	*1.70E−03*
High-fiber diet	All	*MI*	0.3179	1.00E−04	1.00E−04	113424	1.41E−10	*6.48E−10*
		*SGA*	0.1117	6.62E−02	6.56E−02	86914	2.66E−01	2.73E−01
		*MR*	0.1274	5.90E−02	2.91E−02	91129	4.77E−02	5.85E−02
	Gut	*MI*	0.4713	1.00E−04	1.00E−04	10495	2.53E−06	*6.31E−06*
		*SGA*	0.2273	3.93E−02	3.78E−02	7782	1.98E−01	2.14E−01
		*MR*	0.2831	7.70E−03	7.10E−03	8384	3.10E−02	*4.41E−02*
	AGORA	*MI*	0.4197	1.00E−04	1.00E−04	44399.5	2.77E−16	*3.81E−15*
		*SGA*	0.1641	1.69E−02	1.71E−02	30795	1.88E−01	2.12E−01
		*MR*	0.2102	1.50E−03	1.90E−03	33887.5	3.59E−03	*5.70E−03*

^#^adjusted *p* values <0.05 were considered significant

### MI, SGA, and MR scores consistently enrich for CRC bacteria

We evaluated the performance of our scores under different conditions and controlled for potentially confounding factors. Results for the different conditions tested are summarized in Table 3 and individual scores are available in Additional file 2: Table S2. We first evaluated if our scores were robust in enriching for CRC bacteria if we tested different subsets of models. The 1544 models used in the results described above were obtained by reconstructing genome-scale metabolic models for bacteria commonly found in the human microbiome and not specifically the human gut. Furthermore, in our analysis so far, CRC enrichment was defined at a genus level while bacterial association to CRC has been investigated at a higher taxonomic resolution (Table 2 and Additional file 2: Table S2). Thus, we investigated whether our scores would still identify CRC bacteria (1) if we only considered GSMMs generated from gut bacteria and (2) if we defined CRC enrichment on a species-/strain-specific level instead of a genus level. For this purpose, we mapped taxonomic marker genes from the bacterial genomes of our database of GSMMs to the same database used to identify CRC enriched bacteria (see [28] and “Methods” section). This allowed us to identify the closest mOTUs for each of our GSMM and evaluate if the same mOTU was also identified in any of the stool samples from the meta-analysis [28]. We then restricted our analysis to bacteria that were found in these samples because we assumed that they represented gut bacteria. Next, these mappings also allowed us to define whether the closest mOTU for each GSMM was found to be consistently enriched in CRC across different studies (adj. *p* < 1.0E−5 and AUC > .50, Additional file 2: Table S2). Within the subset of human gut bacteria, i.e., those that were identified in stool metagenomes, we found that mOTUs enriched in CRC across studies are also enriched by the MI, SGA, and MR scores (Table 3). Together, these results indicate that the observed response of CRC bacteria to CRC metabolites was not confounded by enrichment for gut bacteria and is still observed at finer taxonomic resolution.

To further corroborate this finding, we tested whether within the gut bacteria, the mOTUs that are depleted in CRC also have significantly lower MI, SGA, and MR scores than the group of enriched mOTUs. Depletion in CRC was defined in more permissive terms than enrichment, since no mOTUs met the significance threshold of adjusted *p* < 1.0E−5 (Additional file 2: Table S2). Instead, we used a cutoff of adjusted *p* < 5.0 E−2. As expected, all three scores were significantly smaller in the group of depleted bacteria compared to the enriched bacteria (*p* = 1.0E−5, *p* = 3.5E−2, and *p* = 6.2E−4, respectively, for the MI, SGA, and MR scores, Mann-Whitney *U* test).

Next, we restricted our analysis only to the subset of models derived from the AGORA study (Additional file 2: Table S2). The models from this study were generated for > 700 bacteria identified as gut isolates [66]. We used this group in an independent test to rule out the possibility that our scores were enriching for gut bacteria rather than for CRC bacteria. Results on this subset and on the subset identified from metagenomes as gut bacteria above were similar to the results on the full database (Table 3, detailed scores are available in Additional file 2: Table S2). These results confirm that the observed enrichment for CRC bacteria was not an indirect effect of enrichment for gut bacteria.

All results described so far were obtained using the basal gut environment predicted by our MAMBO algorithm (see “Methods” section and ref [65]). We evaluated if the choice of alternative in sillico metabolic environments would provide similar results. For this purpose, we used two alternative basal environments derived from the AGORA study [66] referred to as the Western diet and the high fiber diet. We reproduced all our in sillico tests with these alternative basal environments instead of the MAMBO environment. For all conditions, the MI score was still significant and showed significant enrichment of CRC bacteria (Table 3). The SGA score no longer showed significant enrichment of CRC bacteria when the alternative diets were used, suggesting that the SGA score depends more strongly on the choice of basal environment than the MI score (Table 3).

## Discussion

### Changes in the CRC metabolome

Colorectal tumors change the local metabolic environment of the intestine. When a tumor forms, the mucosal barrier becomes impaired, allowing metabolites to diffuse into the intestinal lumen. The change in metabolite composition and reduced mucosal barrier allows opportunistic pathogens to colonize tumor sites in some cases leading to secondary infections and sepsis [11, 68]. For example, the opportunistic bacterium *Streptococcus gallolyticus* subsp. *gallolyticus* causes infections in CRC-patients [68], potentially due to growth advantages at the tumor site [69] and a specific subset of virulence factors [70]. Other site-specific alterations in the CRC tumor-site include changes driven by inflammation and by the Warburg metabolism that causes shifts in pH and oxygen concentration in tumors relative to normal mucosal tissue [71].

### Modeling metabolite response of CRC bacteria

These shifts in the tumor microenvironment facilitate the outgrowth of CRC passenger bacteria, contributing to the assembly of a specific CRC tumor microbiome [11, 72, 73]. Although many factors contribute to the specific CRC tumor microbiome, the metabolome was predicted to be a dominant factor that may account for many of the observed shifts in microbiome community profiles [9]. We have previously shown that the microbial abundances in four different human body sites can be linked to the environmental metabolome by in silico metabolic modeling [65]. Here, we extended our modeling approach and showed that the modeled metabolic capacity of bacteria can be used to predict their specific response to metabolic changes in the environment. To do this, we developed three different scores to quantify the effect of specific metabolites on bacterial growth, that exploit GSMMs of different bacteria. We show that these scores significantly prioritize GSMMs of CRC bacteria over non-CRC bacteria, suggesting that the responses to tumor-associated metabolites explain persistent differences in the gut microbiome of CRC patients relative to healthy controls. In the present study, we only associated bacterial response to metabolites that have been found to be enriched in CRC, since these were by far the most representative set of metabolites. The only metabolites that were found by 3 or more studies to be depleted in CRC were glutamine, glucose, and myoinositol (Table 1) and we thus could not produce meaningful comparisons with metabolite depletion as we did with the 26 CRC enriched metabolites.

### Bacterial drivers and passengers of CRC

As defined in 2012, CRC passengers are bacteria that respond to changes in the tumor environment and are thus enriched in CRC tumor tissue [11]. CRC drivers are bacteria that possess specific oncogenic properties that may drive tumorigenesis. Examples include Enterotoxigenic *Bacteroides fragilis* (ETBF) that is able to degrade and colonize the mucus layer, causing inflammation and increased cell proliferation and colibactin-producing *Escherichia coli* that can cause double-strand breaks in DNA (reviewed in [74–76]). While the current analysis identified CRC passengers, we cannot draw any conclusions about CRC drivers. In fact, some of the passenger bacteria detected herein have been shown to contain mechanisms that drive tumorigenesis, or at least have a role in preparing and sustaining their own niches. On the one hand, *Fusobacterium nucleatum* is among the bacteria that specifically benefit from CRC metabolites. On the other hand, Fusobacterium is also hypothesized to drive tumorigenesis via its unique adhesion protein (FadA) binding to E-cadherin and activating beta-catenin signaling which in turn regulates inflammatory and potentially oncogenic responses. In our current analysis, *F. nucleatum* are among the bacteria that most strongly benefit from the CRC metabolites and may thus be regarded as “driving passengers” [77]. Apart from a few described examples, further research is needed to chart the mechanisms allowing the different constituents of the human microbiome to promote tumor initiation and progression.

### Our general method can be used in other environments

We developed three different scores that integrate GSMMs with lists of metabolites to quantify the effect of specific metabolite enrichment on bacterial growth. Our results show that these scores are able to identify which bacteria respond to the metabolic change. As such, the metabolite importance (MI score), specific growth advantage (SGA score), and metabolite response (MR score) can be applied to answer similar questions in other biomes. It should be noted that our analysis was only possible because we obtained and carefully curated lists of CRC-associated metabolites (Table 1) and bacteria (Table 2). Moreover, we exploited a comprehensive database of > 1500 quality GSMMs from the human microbiome that we developed previously [65]. We obtained better results particularly for the SGA score when using a basal growth environment that was predicted from stool metagenome abundance profiles [65] compared to environments predicted from general diets [66]. While these prerequisites may be difficult to obtain for highly under-sampled environmental biomes, questions about the effect of metabolites on the microbiome in the human system may be more readily answered using our setup. For this reason, we have made a significant effort to make our methods accessible with a detailed online instruction guide, provided as an ipython notebook that contains the information to fully reproduce our results and apply the method to similar systems (see “Methods” section).

Our prediction of CRC passengers proved to be consistent with metagenomic enrichment data and is not incompatible with many of the other aforementioned specific mechanisms that explain the relation of individual bacteria with CRC. A possible future extension could be to include quantitative information about microbes and metabolite abundances, rather than the qualitative, binary classification that we used here (i.e., bacteria and metabolites are CRC-associated or not). In the present study, we integrated information from multiple publications and thus could only provide qualitative definitions of enriched metabolites and bacteria. Nevertheless, the highly significant detection of specific CRC bacteria (Fig. 4) suggests that our approach could also be applied to microbiome studies where quantitative metagenomic and metabolomic data were measured.

## Conclusion

In this study, we have shown that our current understanding of bacterial metabolism, based on genome annotations, allows us to explain the association of bacterial passengers to CRC as being driven by the availability of specific CRC metabolites. Thus, our models and computational experiments suggest that metabolic alterations in the cancer environment are a major component in shaping the CRC microbiome. Our method allowed us to identify likely CRC metabolic passengers which are consistent with experimental studies and indicated that most of the CRC enriched genera are also favored specifically by CRC metabolites and the CRC tumor-like metabolic environment. Beyond the specific question of CRC metabolic passengers, we have provided an example of the systematic use of GSMMs to predict and understand the microbial abundance patterns that are measured by metagenomics, by using mechanistic models that link bacterial metabolism to their metabolic environment.

## Methods

### Genome-scale metabolic models

We used a database consisting of 1544 GSMMs of human-associated microbes from our MAMBO study [65] that includes 763 AGORA human gut GSMMs [66] (Additional file 2: Table S2). These models were built using the ModelSEED pipeline [78] and were tested by flux balance analysis (FBA) [79]. In our previous study [65], gene annotations were used to predict the metabolic reactions that were encoded by each genome. Here, these metabolic reactions were represented by their stoichiometric coefficients in a matrix (**S**) exhibiting reactions as columns and metabolites as rows. The null-space of **S** (**Sv**=**0**) was used as a proxy for the equilibrium reaction rates (**v**), and because **S** does not have a unique solution, specific values of **v** were determined by maximizing a biomass reaction (z) by linear programming. To assure that each model could effectively produce biomass, parsimonious gap-filling was used and a minimal set of reactions that were potentially missing from the models were included.

### CRC metabolites

To identify enriched or depleted metabolites in the tumor sites of CRC patients, we surveyed metabolomics literature. We identified publications with experimental data cited in a review about metabolomics of CRC [80] and additionally reviewed more recent publications. In total. we evaluated 35 publications that mentioned metabolomics and CRC in the abstract and manually inspected these studies for lists of metabolites that were measured in tumor and healthy tissue [30–64]. We found 29 metabolites to be reported as differentially abundant in tumor vs. healthy tissue and present as such in 3 or more publications (Table 1). We used the enriched metabolites to define the CRC tumor microenvironment.

### Basal gut environment

For all experiments described in the main text, we used a basal gut environment predicted by our MAMBO algorithm based on 39 stool metagenomes [65]. This environment was used as proxy for the metabolite concentration that is available for bacteria in the colon and rectal lumina and is defined in terms of relative uptake-rate limits for GSMMs in mmol.gDW^−1^.h^−1^. Additionally, we tested two other basal environments representing proxy for the metabolic composition of the Western diet and high-fiber diet [66]. The formulation of the basal environments is available in Additional file 1: Table S1.

### Importance of CRC metabolites

To rank bacteria by their dependence on CRC metabolites, we defined a metabolite importance score (MI). For this purpose, we first simulated the growth of each GSMMs in the basal environment (obtaining the basal biomass flux z) and then removed each of the basal environment metabolites by blocking their import reactions in the model, leading to a new biomass flux z’. If the growth effect z’/z for a given GSMM fell below a threshold value 0.3; i.e., a more than 70% reduction in predicted growth rate (other threshold values yielded similar results, not shown), the metabolite was considered important for the GSMM. For each GSMM, this resulted in a binary vector containing one component for each metabolite present in the basal diet. This was given the value of 1 if the metabolite was important (i.e., removal decreased growth) or 0 otherwise (Additional file 3: Table S3). These vectors were compared to the CRC metabolites (Table 1) using the Ochiai coefficient [67], resulting in a MI score that we used to rank all bacterial GSMMs. High-ranking bacteria depended strongly on CRC metabolites, and we interpreted these bacteria as potential CRC passengers.

### Growth benefit on CRC metabolites

Next, we evaluated whether bacterial strains responded to the increased availability of the combination of all 26 CRC metabolites in their environment simultaneously. Because GSMMs generally show enhanced growth rates in richer environments, we first created an expected null-distribution of growth responses upon the addition of random metabolites. To do this, we selected one thousand random sets of 26 metabolites from the basal environment and changed their uptake rates to virtually unconstrained values (10^4^ mmol.gDW^−1^.h^−1^). Each time, we compared the new biomass flux z(random) to the biomass flux after supplementing the GSMM with 26 unconstrained CRC metabolites z(CRC). This allowed us to calculate a specific growth advantage score (SGA) defined as the proportion of randomizations whose z(random) was inferior to z(CRC). Finally, all bacteria were ranked by this SGA-score, and the bacteria at the top of this list were interpreted as exhibiting a growth benefit that is specific to CRC-like conditions.

### Combined score

Both the MI and SGA scores provided scores between 0 and 1. We combined both scores into a summarized score that accounts for possible statistical dependence between the scores, we refer to this score as the metabolite response score (MR). For this purpose, we used the Ali-Mikhail-Haq copula function [81], which accounts for the correlation between the two scores within the range that we observed (see “Results” section).

### Enrichment of CRC-associated bacteria

In order to identify bacterial species that are differentially abundant in CRC patients compared to healthy controls, we integrated data from five metagenomic case-control studies [24–28]. For consistency in the bioinformatic analysis, raw sequence data were jointly quality controlled and taxonomically profiled using the mOTU profiler version 2 [82, 83]. Read counts were transformed into relative abundances to account for library size differences between samples. Microbial species that were not detected consistently (maximum relative abundance not exceeding 10^−3^ in at least 3 studies) and the fraction of unmapped reads were discarded. Significance of differential abundance was then tested for each remaining species using a non-parametric permutation-based Wilcoxon test that was blocked for study (and in the case of [26] also for additional metadata indicating sampling before or after diagnostic colonoscopy) as implemented in the R coin package [84]. This blocked test accounts for differences between studies (e.g., due to different DNA extraction protocols or geographic differences in microbiome composition) by estimating the significance based on permutations of the observed data within each block.

For a comprehensive analysis, we unified this list to genus level (Table 2) since this was the lowest taxonomic level that we could unambiguously match species and mOTUs found by metagenomics to be enriched in CRC and the strains for which we had GSMMs. We further attempted to classify our strains using the same set of marker genes that was used to profile metagenomic samples. Each strain was assigned to its closest mOTU present in the mOTU profiler version 2 database [82, 83]. We repeated the experiments using mOTU level classification instead of genus-level classification with the mOTUs that were possible to match with bacterial species identified in the metagenome analysis. Results are reported in the main text as the subset formed by gut bacteria (Table 3).

### Significance of ranking

To assess the significant enrichment of measured CRC bacteria among the ranked lists, we used an approach similar to gene-set enrichment analysis [85, 86]. Briefly, we generated a cumulative weight distribution (W), which was defined by as the normalized fraction of positives minus the fraction of negatives observed in a list, versus the position in the list. High values are obtained if all positives are observed early in the list, in which case the fraction of positives approaches 1 before negatives are seen. Positives were defined as GSMMs of bacteria that were found to be enriched in CRC, negatives were all the other bacteria. We summarized W by its maximum value and used Monte Carlo simulations to assess the likelihood of obtaining max(*W*) by chance. To evaluate if max(*W*) is significant, we generated two empirical null distributions by (i) reshuffling the order of bacteria ten thousand times and (ii) selecting 10,000 random subsets of 13 genera from our bacteria database weighted by the number of species in each genus while keeping the ranked lists in order. For the lists ranked by the metabolite overlap and biomass fold-change scores, we computed empirical *p* values for both null hypotheses (Fig. 4).

### Data availability

All the data used in this study and raw results used in generating the tables and figures are made available at https://github.com/danielriosgarza/bacterial_passengers.py. Additionally, we provide a detailed Ipython notebook that contains the scripts used in this study as well as a thorough explanation of the computational methods we used. This script can be accessed from the GitHub repository and can be used to reproduce all data figures and tables.

## Supplementary information

**Additional file 1: Table S1.** MAMBO, Western diet, and high-fiber diet basal environment.

**Additional file 2: Table S2.** MI, SGA, MR scores, CRC enrichment p-values, AUC, and mOTU prediction for all GSMMs.

**Additional file 3 Table S3.** Important metabolites for GSMMs.

## Data Availability

All the data used in this study and raw results used in generating the tables and figures are made available at https://github.com/danielriosgarza/bacterial_passengers.py.

## Abbreviations

AGORA: Assembly of gut organisms through reconstruction and analysis
AUC: Area under the curve
B: Colorectal cancer
ETBF: Enterotoxigenic *Bacteroides fragilis*
GSMM: Genome-scale metabolic model
MAMBO: Metabolomic analysis of metagenomes using flux balance analysis and optimization
MI: Metabolite importance score
mOTU: Molecular operational taxonomic unit
MR: Metabolite response score
SGA: Specific growth advantage score
